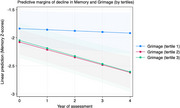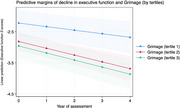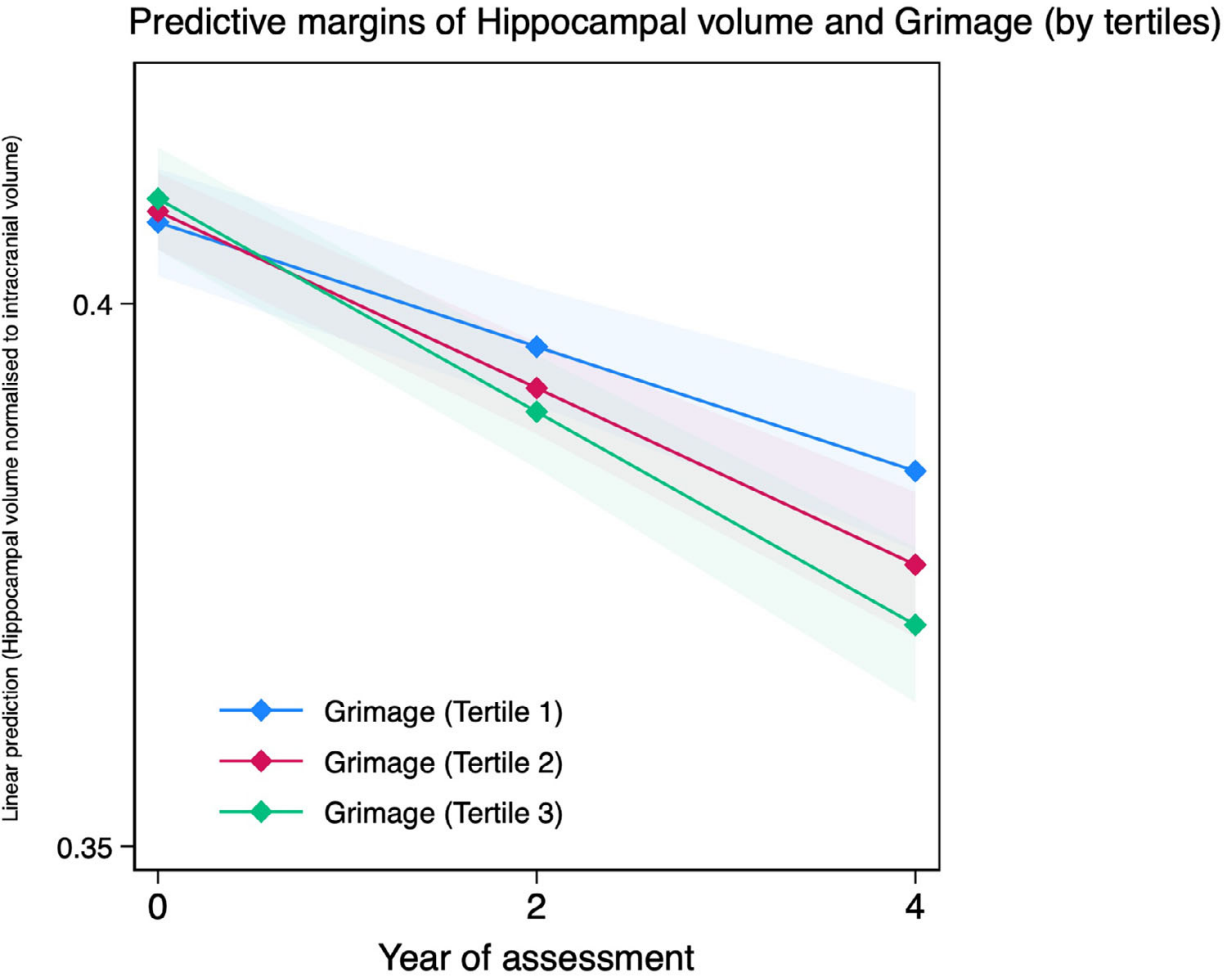# Association of blood‐based DNA methylation measures of biological aging with cognitive decline and hippocampal atrophy

**DOI:** 10.1002/alz70856_105492

**Published:** 2026-01-09

**Authors:** Ming Ann Sim, Yuan Cai, Jian Hua Tay, Rajkumar Dorajoo, Andrea B. Maier, Christopher Chen

**Affiliations:** ^1^ National University of Singapore, Singapore, Singapore, Singapore; ^2^ National University Hospital, Singapore, Singapore, Singapore; ^3^ Lau Tat‐chuen Research Centre of Brain Degenerative Diseases in Chinese, Gerald Choa Neuroscience Institute, Therese Pei Fong Chow Research Centre for Prevention of Dementia, The Chinese University of Hong Kong, Prince of Wales Hospital, Hong Kong SAR, Hong Kong; ^4^ National University of Singapore, Healthy Longevity Translational Research Program, Singapore, Singapore, Singapore; ^5^ Genome Institute of Singapore, Agency for Science, Technology and Research, Singapore, Singapore, Singapore, Singapore; ^6^ National University of Singapore, Singapore, Singapore; ^7^ The Royal Melbourne Hospital, Melbourne, Australia; ^8^ National University Health System, Singapore, Singapore; ^9^ National University Health System, NUHS, Singapore, Singapore, Singapore; ^10^ Memory, Ageing and Cognition Centre, National University Health System, Singapore, Singapore; ^11^ Yong Loo Lin School of Medicine, National University of Singapore, Singapore, Singapore

## Abstract

**Background:**

Processes related to biological aging may underpin dementia pathophysiology. Deoxyribonucleic acid methylation (DNAm) clocks have been positioned as estimators of biological age. However, the predictive ability of blood‐based DNAm biomarkers of aging for cognitive decline and brain atrophy, remains under‐investigated.

**Method:**

A Singaporean memory clinic cohort was examined with a follow‐up for 4 years. Serial cognitive tests of memory and executive function (expressed as Z‐scores) was performed at baseline, and yearly for up to 4 years. Baseline and 2‐yearly brain magnetic resonance imaging (MRI) scans were performed, and volumetric measurements of hippocampal volume were obtained.

DNAm was measured using Epic Illumina on whole blood samples collected at baseline. DNAm age was subsequently calculated using 5 DNAm aging clock algorithms: Horvath1, Horvath2, Hannum, Phenoage, and Grimage, expressed as Z‐scores for analysis.

Longitudinal associations of DNAm age with decline in memory Z‐scores, executive function Z‐scores and hippocampal volume (normalized to intracranial volume) were evaluated using multivariable linear mixed effects models.

**Results:**

Of 300 subjects included (mean age 73.3±7.5 years, 55% female, 53% cerebrovascular disease), 15% had no cognitive impairment, 42% had cognitive impairment no dementia, and 43% had dementia.

DNAm age was associated with faster rates of memory decline: Grimage (β ‐0.051, 95% C.I. ‐0.081, ‐0.021, *p* = 0.001), Phenoage (β ‐0.042, 95% C.I. ‐0.072, ‐0.012, *p* = 0.005), Hannum (β ‐0.039, 95% C.I. ‐0.069, ‐0.0089, *p* = 0.011), Horvath2 (β ‐0.042, 95% C.I. ‐0.072, ‐0.011, *p* = 0.007), Horvath1 (β ‐0.037, 95% C.I. ‐0.068, ‐0.006, *p* = 0.018). Of the DNAm clocks evaluated, only Grimage predicted the rate of decline in Executive function (β ‐0.047, 95% C.I. ‐0.09, ‐0.0024, *p* = 0.039, Figure 1).

All DNAm clocks predicted greater rate of hippocampal atrophy: Grimage (β ‐0.0033, 95% C.I. ‐0.0052, ‐0.0015, *p* <0.001), Phenoage (β ‐0.0035, 95% C.I. ‐0.0053, ‐0.0017, *p* <0.001), Hannum (β ‐0.0037, 95% C.I. ‐0.0055, ‐0.0019, *p* <0.001), Horvath2 (β ‐0.0036, 95% C.I. ‐0.0055, ‐0.0018, *p* <0.001), and Horvath1 (β ‐0.0037, 95% C.I. –0.0056, ‐0.0019, *p* <0.001).

**Conclusion:**

Older blood‐based DNAm age is associated with increased rate of cognitive decline and hippocampal atrophy. Mechanistic studies are required to elucidate the pathobiological underpinnings of DNAm aging with cognitive outcomes.